# Retraction Note: Mitochondrial mechanotransduction through MIEF1 coordinates the nuclear response to forces

**DOI:** 10.1038/s41556-025-01734-6

**Published:** 2025-07-16

**Authors:** Patrizia Romani, Giada Benedetti, Martina Cusan, Mattia Arboit, Carmine Cirillo, Xi Wu, Georgia Rouni, Vassiliki Kostourou, Mariaceleste Aragona, Costanza Giampietro, Paolo Grumati, Graziano Martello, Sirio Dupont

**Affiliations:** 1 Department of Molecular Medicine, University of Padova, Padova, Italy; 2 Department of Biology, University of Padova, Padova, Italy; 3 Telethon Institute of Genetics and Medicine, Pozzuoli, Italy; 4 Department of Mechanical and Process Engineering, ETH Zurich, Zurich, Switzerland; 5 Institute for Bioinnovation, Biomedical Sciences Research Centre “Alexander Fleming”, Athens, Greece; 6 Novo Nordisk Foundation Center for Stem Cell Medicine (ReNEW), University of Copenhagen, Copenhagen, Denmark; 7 Swiss Federal Laboratories for Materials Science and Technology, Dübendorf, Switzerland; 8 Department of Clinical Medicine and Surgery, Federico II University, Naples, Italy

**Keywords:** Mechanotransduction, HIPPO signalling, Lipids, Mitochondria

Retraction to: *Nature Cell Biology* (2024) 26:2046–2060 10.1038/s41556-024-01527-3

The authors have retracted this article. After publication, errors were identified in the images from the microscopy experiments shown throughout the figures (image overlap in Figs. 2bdaf, 4k, 4i, 5fh, and Extended Data Figs. ED3m, ED7ab), and among source data files (data for figures 4, 5, 8, and ED7).

The authors have examined their data files and provided the data to the editors. The authors concluded that source data errors occurred during data transfer between software. Due to the extent of these errors, the authors have decided to retract the article. The authors have been offered to submit a revised manuscript for further peer review.

All authors agree to this retraction.

